# A Rare Concomitant Occurrence of Talon Cusp in Fused Mandibular Permanent Teeth: Report of Two Cases

**DOI:** 10.5005/jp-journals-10005-1437

**Published:** 2017-06-01

**Authors:** Mridula Goswami, Babita Jangra

**Affiliations:** 1 Professor and Head, Department of Pedodontics and Preventive Dentistry, Maulana Azad Institute of Dental Sciences, New Delhi, India; 2 Postgraduate Student, Department of Pedodontics and Preventive Dentistry, Maulana Azad Institute of Dental Sciences, New Delhi, India

**Keywords:** Fusion, Mandibular permanent teeth, Talon cusp.

## Abstract

Fusions of teeth and talon cusp are two rare developmental dental anomalies, affecting both primary and permanent dentitions. Both the anomalies are mostly reported as isolated finding and are rarely associated with other dental anomalies. Talon cusp is more common in maxillary anterior teeth in permanent dentition and fusion is commonly reported in mandibular teeth in primary dentition. The concomitant occurrence of talon cusp and fusion is rare and its occurrence in fused mandibular permanent teeth is also rare. This case report presents two unusual cases of concomitant occurrence of talon cusp with fusion in permanent mandibular incisors. The clinical problems associated with these dental anomalies and importance of their management are discussed in these case reports.

**How to cite this article:**

Goswami M, Jangra B. A Rare Concomitant Occurrence of Talon Cusp in Fused Mandibular Permanent Teeth: Report of Two Cases. Int J Clin Pediatr Dent 2017;10(2):208-212.

## INTRODUCTION

A talon cusp is a horn-like structure projecting from the cingulum area of the maxillary or mandibular anterior teeth in both primary and permanent dentition.^1-3^ Its prevalence rate varies from 0.04 to 10% in the English literature.^4^ Males show higher incidence as compared with females.^4^ Permanent dentition is commonly involved as compared with primary dentition and often seen in maxillary lateral (55%) or central (33%) incisors followed by mandibular incisors (6%) and maxillary canine (4%).^3,4^ Talon cusp in mandibular anterior teeth is very rare and concomitant occurrence of its fusion is still rarer.^4^ This study reports two unusual cases of talon cusp-infused mandibular permanent incisors.

## CASE REPORTS

### Case 1

**Fig. 1:**
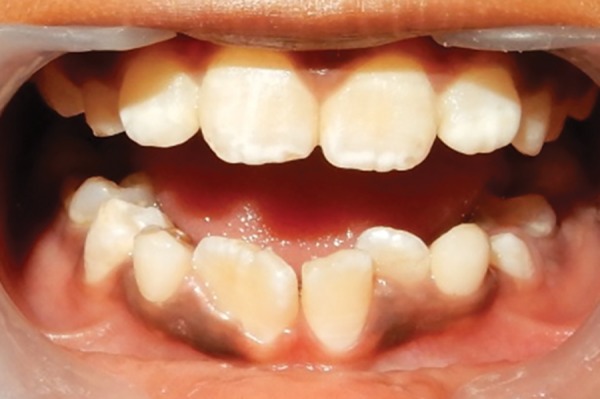
Labial view showing fusion between permanent mandibular right central and lateral incisors (41 and 42)

**Fig. 2:**
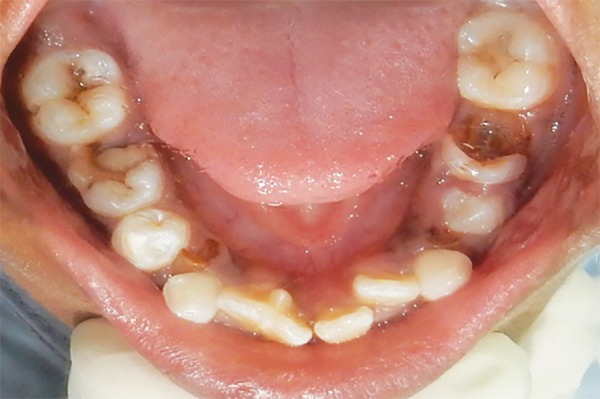
Mandibular occlusal view showing talon cusp on lingual surface of fused 41 and 42

**Fig. 3:**
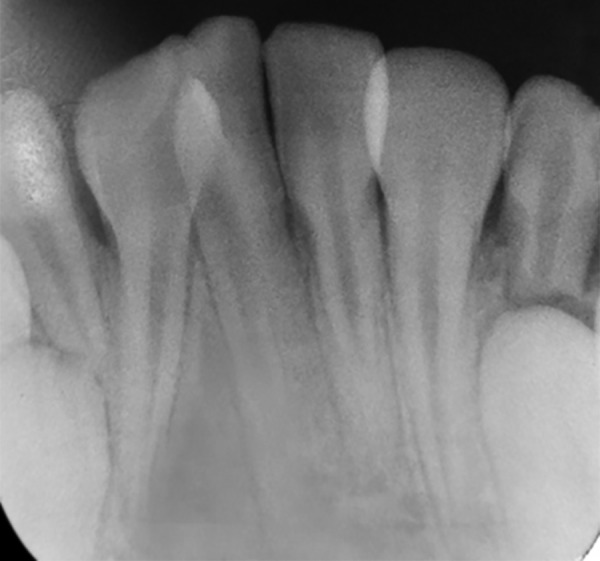
Intraoral periapical radiograph showing fusion of crowns with two separate roots with respect to 41 and 42 with lingual talon cusp

**Fig. 4:**
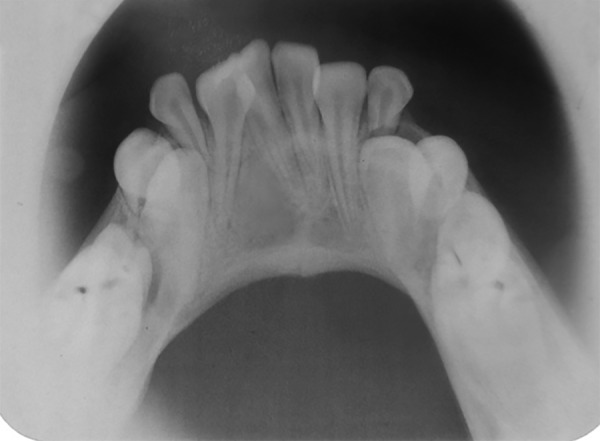
Mandibular occlusal view radiograph showing fusion of mandibular teeth with talon cusp with respect to 41 and 42

**Fig. 5:**
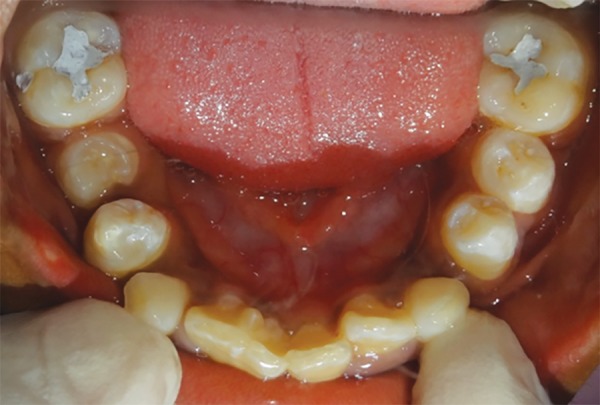
Mandibular occlusal view showing restored teeth 36, 46 and restored fused teeth 41 and 42

A 10-year-old male patient reported to the Department of Pedodontics and Preventive Dentistry with his mother, with a chief complaint of pain in the left lower back tooth region since 7 days. The pain was dull and intermittent in nature. No significant medical and family history was obtained. There was no significant past dental history and no history of orofacial trauma. On general physical examination, patient was apparently well. On intraoral examination, left and right mandibular permanent first molars were carious and left and right mandibular primary second molars were grossly decayed. Root stumps of left and right mandibular primary first molars were present. In maxillary arch, left and right primary second molars were grossly decayed. During intraoral examination, it was found that permanent mandibular right central incisor had a large crown with horn-like projection on the lingual surface of tooth (Fig. 1). The permanent mandibular right central and lateral incisors were provisionally diagnosed to be fused as lateral incisors were not visible in the oral cavity. Two-thirds part of the crown of tooth was erupted in oral cavity and was rotated from its normal position (Fig. 2). Intraoral periapical (Fig. 3) and mandibular occlusal radiographs were taken (Fig. 4). Both the radiographs revealed fusion of only crown of permanent mandibular right central and lateral incisor teeth with two separate roots. Fused teeth showed two pulp chambers with two root canals. An inverted “V“-shaped radiopaque structure arising from the cingulum of teeth superimposed on affected crowns of teeth. This horn-like projection consisted of enamel and dentin with pulp chamber, which confirmed the presence of a talon cusp. After examination and confirmation of diagnosis of all the carious teeth, necessary treatment was rendered to the child. The fused teeth were clinically asymptomatic and the talon cusp neither irritated the tongue during speech or mastication nor did it interfere with occlusion. As there was no clinical problem associated with the fused teeth, clinical findings were explained as both parents and patient were unaware of the related condition, reassurance of the patient was done, and pit and fissure sealants were applied on the fused teeth to prevent any decay or further problem. Patient was scheduled for extraction of grossly carious teeth and root stumps. All the carious teeth were restored (Fig. 5). Patient was kept on regular follow-up for examination of fused teeth.

### Case 2

**Fig. 6:**
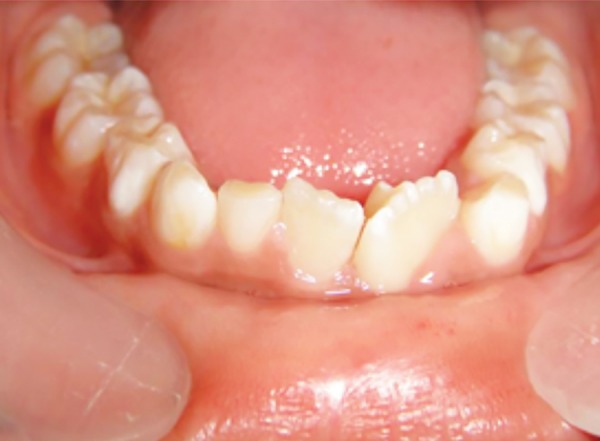
Labial view showing fusion between permanent mandibular left central and lateral incisors (31 and 32)

**Fig. 7:**
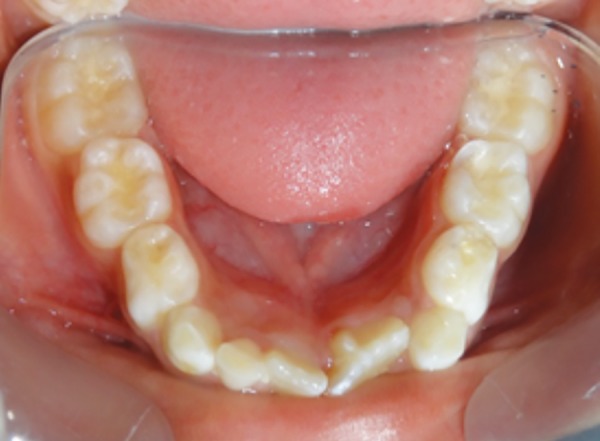
Mandibular occlusal view showing talons cusp on lingual surface of fused 31 and 32

**Fig. 8:**
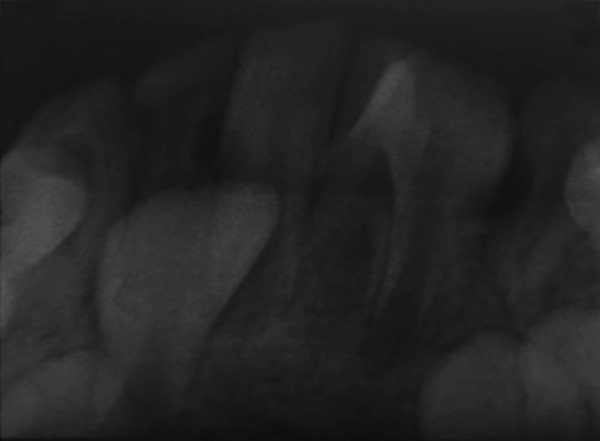
Intraoral periapical radiograph showing fusion of crowns with two separate roots with respect to 31 and 32 with lingual talon cusp

**Fig. 9:**
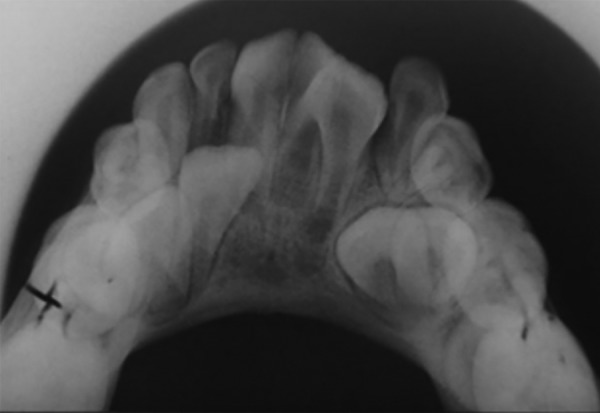
Mandibular occlusal view radiograph showing fusion of mandibular teeth with talon cusp with respect to 31 and 32

**Fig. 10:**
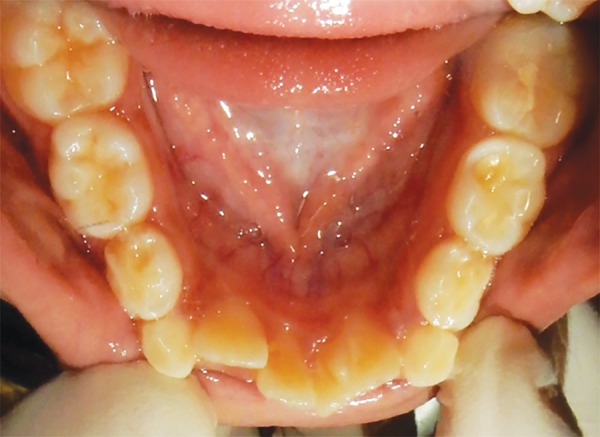
Mandibular occlusal view showing restored teeth 36 and fused teeth 31 and 32

A 6-year-old male patient reported to the Department of Pedodontics and Preventive Dentistry with his mother, with a chief complaint of large, unsightly mandibular central incisor. Patient’s medical and family history was noncontributory. There was no significant past dental history and no history of orofacial trauma. On extra-oral examination, no clinical abnormality was found. On intraoral examination, permanent mandibular left central incisor had a large crown (Fig. 6). The tooth was erupting in the oral cavity with distal rotation from its normal position. The permanent mandibular left central and lateral incisors were provisionally diagnosed to be fused as lateral incisor was not visible in the oral cavity. A pyramidal cusp-like projection resembling the talon cusp was observed on the lingual surface of tooth (Fig. 7). Intraoral periapical (Fig. 8) and mandibular occlusal radiographs (Fig. 9) were taken, which revealed fusion of teeth on the coronal aspect, and two separate roots in close apposition with each other. Both the radiographs revealed the fusion of crowns of the two teeth with two pulp chambers, two root canals with open apex, and inverted “V“-shaped radiopaque structure superimposed on affected crowns. The inverted “V“-shaped structure consisted of enamel and dentin with pulp chamber arising from the cingulum of teeth, which confirmed the presence of a talon cusp. Reassurance of the patient was done, and pit and fissure sealants were applied on the fused teeth to prevent any decay or further problem. Patient was scheduled for periodic dental examination (Fig. 10).

## DISCUSSION

A talon cusp is an odontogenic anomaly with unknown etiology.^4,5^ Its association is seen with other anomalies like peg-shaped lateral incisor, impacted canines, mesiodens, complex odontomas, dens evaginatus of posterior teeth, shovel-shaped incisors, dens invaginatus, and exaggerated carabelli cusp.^6,7^

Fusion is the union of two adjacent teeth at the crown level.^8,9^ It is common in primary dentition and frequently involves mandibular lateral incisor and canine.^8,9^ It may be associated with syndromes like achondroplasia, chondroectodermal dysplasia, focal dermal hypoplasia, and osteopetrosis.^10^ The genetic predisposition and environmental factors, such as thalidomide embryopathy, fetal alcohol exposure, or hypervitaminosis A of the pregnant mother can affect development of tooth.^10^

The unique occurrence of fusion and talon cusp in the same tooth (presented in these case reports) indicates that local hyperactivity of the dental lamina or its remnants may continue from the bud stage to morphodifferentia-tion.^7^ In the literature, only a few previous case reports of talon cusp with fusion in the mandibular incisors are reported.^5,8-12^ Li^5^ reported one case of bilateral double teeth associated with a talon cusp. Ekambaram et al^8^ reported one case of fusion in the mandibular permanent incisors with facial and lingual talon cusps. Prabhakar et al^9^ reported one case of bilateral fusion of permanent mandibular incisors with talon cusp. Rao and Hegde^10^ reported one case of fusion in mandibular permanent incisors with alingual talon cusp. Thirumalaisamy et al^11^ reported one case of talon cusp on fused permanent mandibular incisors. Cho^12^ reported two cases of talon cusp in permanent mandibular anterior teeth with fusion. Sharma and Nagpal^13^ reported one talon cusp on a fused permanent mandibular incisor. A similar of case of talon cusp on a fused permanent mandibular incisor was reported previously at the Department of Pedodontics and Preventive Dentistry.^14^ The prevalence rates have been reported to be 0.6% in Chinese, 0.9% in Japanese, 2.4% in Jordanians, and 5.2% in Malaysians.^12^ The incidence is 1 to 2% in Asian communities^15^ and between 3 and 4% in Eskimos and North American Indians.^16^

It may be associated with increased caries susceptibility, interference of tongue during speech and mastication, compromised esthetics, occlusal interference, and accidental cusp fracture and attrition.^7-12^ It can lead to misinterpretation of radiographs of involved teeth before eruption.^11^

Chawla et al^17^ classified talon cusp into three types, namely true, semi, and trace talon.

Type I or true talon is a morphologically well-delineated additional cusp that prominently projects from the palatal surface of a primary or permanent anterior tooth and extends to at least half the distance from the cementoenamel junction to the incisal edge.

Type II or semi talon is an additional cusp of 1 mm or more that extends to less than half the distance from the cementoenamel junction to the incisal edge and blends with the palatal surface or stands away from the crown.

Type III or trace talon is an enlarged or prominent cingulum with variations, such as conical, bifid, or tubercle like.

In the present case report, both the cases were diagnosed with type I talon cusp.

Tannenbaum and Alling^18^ described the classification of fusion and differentiated it with term gemination and twinning diagrammatically. Classification is as follows:

- Gemination is cleavage of a single tooth germ.
  – Partial cleavage (true gemination)
  – Complete cleavage (twinning).

Fusion occurs when two separate tooth germs fused during formative stage.

- Union by enamel and dentin (true fusion)
- Union by dentin and/or cementum (late fusion); a late fusion by cementum is called a concrescence. In this present case report, both the cases were diagnosed with true fusion.

Talon cusp in mandibular anterior teeth is very rare, and concomitant occurrence of talon cusp in mandibular anterior teeth with development anomalies, i.e., fusion, is very rare. To the best of our knowledge, there are only eight case reports who reported talon cusp in fused man-dibular anterior teeth (as per Google search). In the present case report, clinical and radiographic examinations make a definite diagnosis of fusion. Clinically, total tooth number in the mandibular arch was less and tooth count revealed a missing tooth.

The treatment of talon cusp includes sealant application in the developmental groove, gradual reduction, followed by topical fluoride application and endodontic treatment in cases which have pulp exposure.^7-12^ In the present cases, as there were no associated problems with involved teeth, prophylactic placement of pit and fissure sealants was done in the developmental groove. If esthetics remains the major concern, then root canal treatment followed by complete crown is indicated for such cases.

## CONCLUSION

Talon cusp is a rare dental anomaly, which is associated with various clinical problems. If it’s association occurs with another dental anomaly like fusion, further exaggeration of problems may occur. A careful monitoring should be done by clinical and radiographic observations to prevent development of any pathology. Further, these cases require long-term follow-up for prevention of any problem in the future.
